# Ventilation liberation in Ibero-American pediatric intensive care units

**DOI:** 10.62675/2965-2774.20240163-en

**Published:** 2024-09-05

**Authors:** José Colleti, Arnaldo Prata-Barbosa, Cristian Tedesco Tonial

**Affiliations:** 1 Hospital Luz Vila Mariana São Paulo SP Brazil Hospital Luz Vila Mariana - São Paulo (SP), Brazil.; 2 Instituto D’Or de Ensino e Pesquisa Rio de Janeiro RJ Brazil Instituto D’Or de Ensino e Pesquisa - Rio de Janeiro (RJ), Brazil.; 3 Universidade Federal do Rio Grande do Sul Hospital de Clínicas de Porto Alegre Porto Alegre RS Brazil Hospital de Clínicas de Porto Alegre, Universidade Federal do Rio Grande do Sul - Porto Alegre (RS), Brazil.

Mechanical ventilation (MV) is a life-saving intervention. Because it is associated with complications, patients should be weaned from the ventilator as soon as the underlying condition that led to MV has sufficiently improved and the patient can safely maintain spontaneous breathing.^(1)^ Despite wide acceptance of a reduced time on MV, there are few studies on the weaning and extubation of pediatric patients. A significant proportion of patients being evaluated for weaning are ready for extubation, suggesting that weaning is often not considered early enough during ventilation.^(1)^

The indications for extubation are often not clear, and several scores have been developed to predict the success of weaning and extubation, but whether such indications and scores are beneficial is unclear.^(2)^ Moreover, the variability of diseases makes the application of these scores difficult. Extubating a patient with obstructive respiratory disease is different from extubating a patient in the postoperative period of cardiac surgery with myocardial dysfunction. New techniques for assessing readiness for weaning and predicting extubation success are being developed but are far from being generally accepted in pediatric practice. While there have been some physiologic, observational, and even randomized controlled trials on aspects of ventilator liberation in pediatric patients, robust research data are lacking. Given the lack of data, an approach that combines a systematic review with the consensus opinion of international experts could generate high-quality recommendations and terminology definitions to guide clinical practice and highlight important areas for future research in weaning, extubation readiness, and liberation from MV following pediatric respiratory failure.

Intensive care unit liberation is an evidence-based bundle associated with improved outcomes in critically ill adults and children.^(1)^ A key part of the Society of Critical Care Medicine ICU liberation bundle is the "B" component, which typically involves extubation readiness tests (ERTs). An ERT commonly includes a spontaneous breathing trial (SBT) and other variables that clinicians use to determine extubation readiness. SBTs decrease the duration of invasive MV, incidence of complications, and cost in mechanically ventilated patients.^(2)^ Although there are limited data on SBTs and ERTs in critically ill children, these tools are important for promoting timely ventilator liberation. Protocols involving the ICU liberation bundle have been implemented in several pediatric ICUs, including standardized methods to assess extubation readiness, often including ERTs, with mixed methodology and success.^(3)^ There is significant variability in the approach and comprehensiveness of ventilator liberation protocols among pediatric ICUs. Some of this variability is related to provider preferences, patient population, and the lack of clear clinical practice guidelines. However, there are likely pediatric ICU-specific resources and characteristics, which may result in unwarranted variation in ventilator liberation practices.

In this context, Retta et al. published a survey in Critical Care Science conducted among 138 Ibero-American pediatric ICUs to identify the use of standardized protocols, analyze the criteria and parameters used during invasive MV liberation, and evaluate the use of noninvasive ventilation (NIV) after extubation. ^(4)^ The authors reported that only 47 of the included pediatric ICUs (34.1%, 47/138) had a written protocol for the liberation of invasive MV. They reported that the elements included in the ERT were sedation and analgesia protocols (66%), criteria for defining ERT failure (66%), a standardized spontaneous ventilation test (57.4%), preestablished criteria for NIV or high-flow nasal cannula (HFNC) postextubation support (40.4%), and an extubation failure checklist (31.9%). Approximately 40.4% of patients did not meet the preestablished criteria for the indication of noninvasive respiratory support. These results are worrying, as clinical practices are not in line with international guidelines for ventilation liberation in the ICU. This study highlights the need to disseminate best clinical practices related to the liberation of MV in Ibero-American countries.

In other worldwide surveys representing 380 pediatric ICUs from 47 different countries and 555 pediatric intensivists, including those in Latin America, Loberger et al. reported that international pediatric ventilation liberation practices are heterogeneous. Protocols for SBTs (50%) and endotracheal tube cuff management (55.8%) were the only protocols used by more than or equal to 50% of the pediatric ICUs, and postextubation respiratory support protocols were not prevalent (28.7%).^(5,6)^ Overall, variability in international pediatric ventilation liberation practices is high, and the prevalence of protocol implementation is generally low.

The first International Clinical Practice Guidelines for Pediatric Ventilator Liberation, supported by Pediatric Acute Lung Injury and Sepsis Investigators (PALISI), sought to develop the first international pediatric-specific ventilator liberation clinical practice guidelines focused on acutely hospitalized children receiving invasive MV for more than 24 hours.^(7)^ Although some observational and interventional studies related to pediatric ventilator liberation exist, most of the pediatric literature is limited to narrative reviews and meta-analyses. The guidelines may reduce the variability of clinical practices, disseminate knowledge of ventilation liberation and inspire new robust studies in the field, for example, to evaluate in which patient profile SBTs and ERTs would be most effective.

The main message is that we need to better understand the barriers to implementing the recommended ventilation liberation practices, including all the ICU liberation bundle components, known as the ABCDEF bundle, which have been adopted with substantial variability across regions.^(8)^

The study by Retta et al.^(4)^ is timely because it sheds light on a problem that appears to be quite prevalent around the world, showing the variability of clinical practices and low adherence to international guidelines on ventilation liberation. We believe that only through continued education and the dissemination of international guidelines, along with robust clinical research, will we achieve adherence to the best clinical practices.
