# Supplementary material for: Ventilation liberation in Ibero-American pediatric intensive care units
Source: Crit Care Sci. 2024 Sep 5;36:e20240163en. doi: 10.62675/2965-2774.20240163-en (PMC11463984; doi:10.62675/2965-2774.20240163-en)
Supplement: Translation

# Translation: Liberação do ventilador em unidades de terapia intensiva pediátrica ibero-americanas

**DOI:** 10.62675/2965-2774.20240163-pt

**Authors:** José Colleti, Arnaldo Prata-Barbosa, Cristian Tedesco Tonial

**Affiliations:** 1 São Paulo SP Brasil Hospital Luz Vila Mariana - São Paulo (SP), Brasil.; 2 Rio de Janeiro RJ Brasil Instituto D’Or de Ensino e Pesquisa - Rio de Janeiro (RJ), Brasil.; 3 Porto Alegre RS Brasil Hospital de Clínicas de Porto Alegre, Universidade Federal do Rio Grande do Sul - Porto Alegre (RS), Brasil.

A ventilação mecânica (VM) é uma intervenção que salva vidas. Como está associada a complicações, os pacientes devem ser liberados do ventilador assim que a condição subjacente que levou à VM tiver melhora suficiente e o paciente puder manter a respiração espontânea com segurança.^(1)^ Apesar da ampla aceitação de um tempo reduzido de VM, há poucos estudos sobre o desmame e a extubação de pacientes pediátricos. Grande parte dos pacientes em avaliação para desmame está pronta para extubação, o que sugere que o desmame, muitas vezes, não é considerado precocemente o suficiente durante a ventilação.^(1)^

As indicações de extubação, em geral, não são claras, e desenvolveram-se vários escores para prever o sucesso do desmame e da extubação, mas não está claro se essas indicações e escores são benéficos.^(2)^ Além disso, a variabilidade das doenças dificulta a aplicação desses escores. A extubação de um paciente com doença respiratória obstrutiva é diferente da de um paciente no pós-operatório de cirurgia cardíaca com disfunção miocárdica. Há novas técnicas sendo desenvolvidas para avaliar a prontidão para a extubação e prever seu sucesso, mas estão longe de serem globalmente aceitas na prática pediátrica. Embora existam alguns estudos fisiológicos, observacionais e até mesmo controlados randomizados sobre aspectos associados à liberação da VM em pacientes pediátricos, faltam estudos com dados robustos. Dada a falta de dados, uma abordagem que combine uma revisão sistemática com a opinião consensual de especialistas internacionais poderia gerar recomendações de alta qualidade e definições de terminologia para orientar a prática clínica e destacar áreas importantes para futuras pesquisas de desmame, prontidão para extubação e liberação da VM após insuficiência respiratória pediátrica.

A liberação da VM na unidade de terapia intensiva é um componente do *bundle* baseado em evidências associado a desfechos favoráveis em adultos e crianças em estado crítico.^(1)^ Uma parte importante do *bundle* de liberação da VM na UTI da *Society of Critical Care Medicine* é o componente "B", que normalmente envolve testes de prontidão de extubação (TPEs). O TPE geralmente inclui um teste de respiração espontânea (TRE) e outras variáveis que os clínicos usam para determinar a prontidão para a extubação. Os TREs reduzem a duração da VM invasiva, a incidência de complicações e o custo em pacientes em VM.^(2)^ Embora existam poucos dados sobre TREs e TPEs em crianças em estado crítico, essas ferramentas são importantes para estimular a retirada oportuna da VM. Várias UTIs pediátricas implementaram protocolos envolvendo o pacote de liberação da VM na UTI, incluindo métodos padronizados para avaliar a prontidão para a extubação, muitas vezes incluindo TPEs, com metodologia e sucesso mistos.^(3)^ Há uma variabilidade significativa na abordagem e na abrangência dos protocolos de liberação da VM em UTIs pediátricas. Parte dessa variabilidade está relacionada às preferências do profissional, à população de pacientes e à falta de diretrizes claras de prática clínica. No entanto, é provável que existam recursos e características específicas da UTI pediátrica, o que pode resultar em variações injustificadas nas práticas de liberação da VM.

Nesse contexto, Retta et al. publicaram na *Critical Care Science* um estudo que incluiu 138 UTIs pediátricas ibero-americanas para identificar o uso de protocolos padronizados, analisar critérios e parâmetros utilizados durante a retirada da VM invasiva e avaliar o uso de ventilação não invasiva (VNI) após a extubação.^(4)^ Os autores relataram que apenas 47 das UTIs pediátricas incluídas (34,1%, 47/138) tinham um protocolo escrito de lilberação de VM invasiva. Eles relataram que os elementos incluídos no TPE eram protocolos de sedação e analgesia (66%), critérios de definição de falha do TPE (66%), um TRE padronizado (57,4%), critérios predefinidos de suporte pós-extubação com VNI ou cânula nasal de alto fluxo (CNAF) (40,4%) e uma lista de verificação de falha de extubação (31,9%). Aproximadamente 40,4% dos pacientes não atenderam aos critérios predefinidos de indicação de VNI. Esses resultados são preocupantes, pois as práticas clínicas não estão em conformidade com as diretrizes internacionais para liberação da VM na UTI. Este estudo destaca a necessidade de disseminar as melhores práticas clínicas relacionadas à liberação da VM nos países ibero-americanos.

Em outro inquérito mundial com 380 UTIs pediátricas de 47 países diferentes e 555 intensivistas pediátricos, incluindo os da América Latina, Loberger et al. relataram que as práticas internacionais de liberação da VM pediátrica são heterogêneas. Protocolos de TREs (50%) e manejo do balonete do tubo endotraqueal (55,8%) foram os únicos protocolos usados a partir de 50% das UTIs pediátricas, e não predominaram (28,7%) protocolos de suporte respiratório pós-extubação.^(5,6)^ Em geral, é alta a variabilidade nas práticas internacionais de liberação da VM pediátrica, e a prevalência de implementação de protocolos é geralmente baixa.

As primeiras Diretrizes Clínicas Internacionais para a Liberação da Ventilação na Pediatria, apoiadas pelo *Pediatric Acute Lung Injury and Sepsis Investigators* (PALISI), buscaram desenvolver as primeiras diretrizes internacionais de prática clínica de liberação da VM específicas na pediatria, voltadas a crianças hospitalizadas em estado agudo que receberam VM invasiva por mais de 24 horas.^(7)^ Embora existam alguns estudos observacionais e intervencionistas relacionados à liberação da VM pediátrica, a maior parte da literatura pediátrica limita-se a revisões narrativas e metanálises. As diretrizes podem reduzir a variabilidade das práticas clínicas, disseminar o conhecimento da liberação da VM e inspirar novos estudos robustos na área, por exemplo, visando avaliar em que perfil de paciente os TREs e os TPEs seriam mais eficazes.

A principal mensagem é que precisamos entender melhor as barreiras à implementação das práticas recomendadas de liberação da VM, incluindo todos os componentes do *bundle* de liberação da VM na UTI, conhecido como *bundle* ABCDEF, adotado com grande variabilidade entre as regiões.^(8)^

O estudo de Retta et al.^(4)^ é oportuno porque lança luz sobre um problema que parece ser bastante prevalente em todo o mundo, mostrando a variabilidade das práticas clínicas e a baixa adesão às diretrizes internacionais de liberação da VM. Acreditamos que somente por meio da educação continuada e da disseminação de diretrizes internacionais, com uma pesquisa clínica robusta, conseguiremos a adesão às melhores práticas clínicas.
